# Data augmented lung cancer prediction framework using the nested case control NLST cohort

**DOI:** 10.3389/fonc.2025.1492758

**Published:** 2025-02-25

**Authors:** Yifan Jiang, Venkata S. K. Manem

**Affiliations:** ^1^ Centre de Recherche du CHU de Québec, Université Laval, Québec, QC, Canada; ^2^ Département de Biologie Moléculaire, Biochimie Médicale et Pathologie, Université Laval, Québec, QC, Canada; ^3^ Institut Universitaire de Cardiologie et de Pneumologie de Québec, Québec, QC, Canada

**Keywords:** data augmentation, lung cancer, cancer risk prediction, computed tomography, Artificial Intelliegnce, machine learning

## Abstract

**Purpose:**

In the context of lung cancer screening, the scarcity of well-labeled medical images poses a significant challenge to implement supervised learning-based deep learning methods. While data augmentation is an effective technique for countering the difficulties caused by insufficient data, it has not been fully explored in the context of lung cancer screening. In this research study, we analyzed the state-of-the-art (SOTA) data augmentation techniques for lung cancer binary prediction.

**Methods:**

To comprehensively evaluate the efficiency of data augmentation approaches, we considered the nested case control National Lung Screening Trial (NLST) cohort comprising of 253 individuals who had the commonly used CT scans without contrast. The CT scans were pre-processed into three-dimensional volumes based on the lung nodule annotations. Subsequently, we evaluated five basic (online) and two generative model-based offline data augmentation methods with ten state-of-the-art (SOTA) 3D deep learning-based lung cancer prediction models.

**Results:**

Our results demonstrated that the performance improvement by data augmentation was highly dependent on approach used. The Cutmix method resulted in the highest average performance improvement across all three metrics: 1.07%, 3.29%, 1.19% for accuracy, F1 score and AUC, respectively. MobileNetV2 with a simple data augmentation approach achieved the best AUC of 0.8719 among all lung cancer predictors, demonstrating a 7.62% improvement compared to baseline. Furthermore, the MED-DDPM data augmentation approach was able to improve prediction performance by rebalancing the training set and adding moderately synthetic data.

**Conclusions:**

The effectiveness of online and offline data augmentation methods were highly sensitive to the prediction model, highlighting the importance of carefully selecting the optimal data augmentation method. Our findings suggest that certain traditional methods can provide more stable and higher performance compared to SOTA online data augmentation approaches. Overall, these results offer meaningful insights for the development and clinical integration of data augmented deep learning tools for lung cancer screening.

## Introduction

Lung cancer remains the leading mortality cause of cancer in the North America. In 2023, an estimated 32,100 new and 20,700 deaths are expected in Canada, which makes lung cancer accountable for approximately 1 in 4 cancer deaths (1, 2). The patients diagnosed with distant metastatic disease (stage IV) had a 1-year survival rate of 15~19% compared with 81-85% for early stage (stage I) (3), which emphasizes the importance and clinical urgency for lung cancer early detection.

Low-dose computed tomography (LDCT) offers lower radiation exposure compared to a standard CT scan, making it an effective tool for regular lung cancer screening to improve early detection and reduce mortality. The American Association for Thoracic Surgery recommend annual lung cancer screening with LDCT for adults aged 55-79 years who have a long-term smoking history and currently smoke or have quit smoking within the past 15 years (4). The National Lung Screening Trial (NLST), a large randomized controlled trial sponsored by the National Cancer Institute (NCI), aimed to identify the efficacy of LDCT in lung cancer screening compared to standard chest X-ray among high-risk patients. It enrolled 53,454 current or former cigarette smokers aged 55 to 74 with at least a 30 pack-year smoking history. The study found a 20% reduction in lung cancer mortality in the LDCT arm compared to the chest X-ray arm during the follow-up period (5).

In this regard, the recent advances in deep learning have greatly encouraged its applications in the medical imaging domain, specifically in cancer diagnoses and treatments (6, 7). Despite remarkable achievements, there are still two major issues hindering further application of deep learning in the medical imaging domain, and their translation to a clinical setting, namely: a) Data shortage: The performance of deep learning-based diagnostic models greatly depends on high-quality data, i.e., a well-labeled and massive dataset can significantly enhance diagnostic capabilities. However, due to patient privacy issues and the time-consuming labeling process, data scarcity is commonly seen in medical imaging areas, significantly limiting the deployment of deep learning models in lung cancer screening (8); b) Imbalanced data: Imbalanced data, where one category is significantly underrepresented compared to the others, can be commonly observed in lung cancer screening trials (malignant cases are much less prevalent than benign cases). In such circumstances, models tend to be biased towards the majority category, resulting in poor diagnostic performance for the minority category. This is because, during training, overfitting is more likely to occur for the minority category due to the limited number of samples available (9).

To overcome the data shortage and imbalanced data problems, data augmentation technologies have been introduced into various computer vision areas, such as image classification, object detection, and segmentation (10, 11). These technologies largely fall into five categories:

1. *Geometric transformations* are operations that change the position, orientation, or scale of a geometric image or shape in a coordinate plane, including rotations, translations, reflections, and dilations.
2. *Photometric transformations* modify pixel intensity without changing the geometric structure of an image, including brightness and contrast adjustment, color space transformation, noise addition, blurring, and sharpening (12).
3. *Mixing images*, also known as image blending or compositing, combines two or more images to create a novel synthetic image. Recently emerged methods include Mixup (13) and CutMix (14).
4. *Random erasing* randomly selects a rectangular region within an input image and erases its pixels by replacing them with random or specific values. It is widely applied in tasks such as image classification (15) and object detection (16).
5. *Generative model-based methods* leverage cutting-edge generative models, such as Generative Adversarial Networks (GANs), Variational Autoencoders (VAEs), and diffusion models, to generate novel synthetic data for augmenting existing datasets (17).

Although data augmentation has become an essential method for training deep learning models in many applications, it has not been comprehensively reviewed in the context of lung cancer detection. In this study, we selected seven typical data augmentation methods from the above categories: geometric data augmentation, RandAugment (12), Mixup, Cutout, CutMix, Med-DDPM (18), and HA-GAN (19). The first five methods were originally designed for 2D image tasks and adjusted to 3D volume tasks in this paper, which are for online data augmentation, meaning they directly participate in the training procedure and augment the dataset in parallel. The latter two approaches are based on generative models (diffusion models and GANs) and perform offline data augmentation, allowing them to generate synthetic samples that can be used to augment the original dataset before the training procedure. In the current work, we performed a systematic comparison of the above seven data augmentation methods on ten different deep learning-based lung cancer predictors. Each predictor was trained and tested on a subset of the NLST dataset, and the results were reported using different metrics. Through the evaluation and analysis of the experiments, we aim to identify a suitable data augmentation method that can be used to develop a highly efficient and a clinically relevant lung cancer screening tool.

## Materials and methods

### Description of cohorts

**Figure 1:**
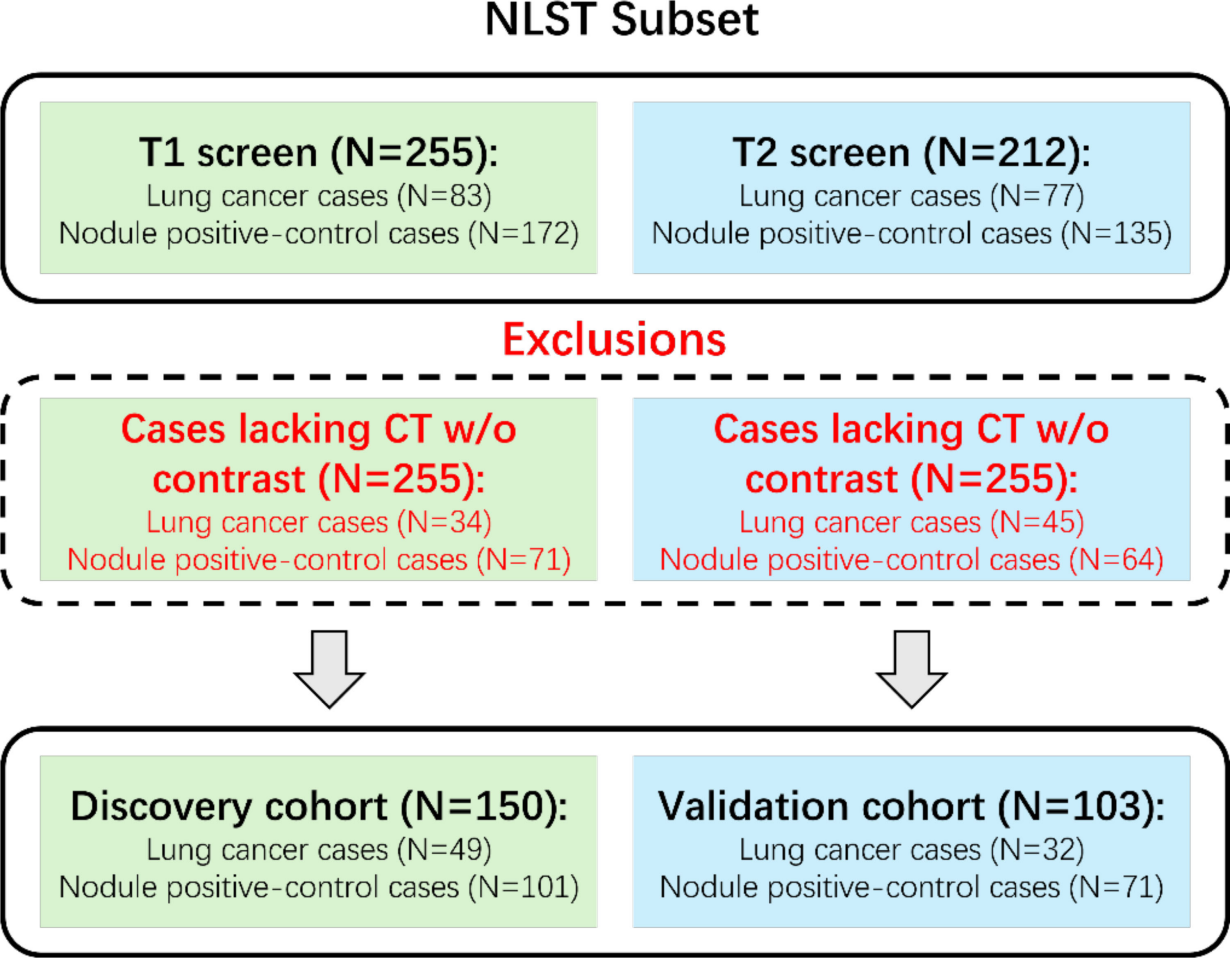
Dataset construction consort diagram.

The National Lung Screening Trial (5) was a large randomized controlled trial that studied the effectiveness of LDCT for early detection of lung cancer in high-risk individuals. 53,454 participants aged 55 to 74 with at least 30 pack-years of smoking history were randomly assigned to either annual LDCT or chest X-ray screening for a 3-year period. Previous works have stated the detailed study design and main findings for the NLST dataset. We were granted access to LDCT images and clinical records through the National Cancer Institute (NCI) Cancer Image Archive. Due to the availability of annotations, we considered the case-control analysis of Cherezov et al. (20) to select 253 participants who had CT scans without contrast. Among these 253 cases, 50 were from the first follow-up screen (T1), and 103 were from the second follow-up screen (T2). Participants from T1 were assigned to the discovery cohort (N=150), and those from T2 were assigned to the validation cohort (N=103) for the evaluation of lung cancer prediction. Note that the discovery cohort was also used for training the generative model-based data augmentation methods. Nodule segmentation annotations were provided by Cherezov et al. (20). To further clarify the data organization, we demonstrate the dataset construction consort diagram of lung cancers and nodule‐positive controls in **Figure 1**.

### Deep learning based lung cancer predictors

To comprehensively evaluate data augmentation methods for lung cancer prediction, we selected 11 representative 3D deep learning models based on their adaptability to 3D applications, prominence in medical imaging analysis, and availability of pretrained models, ensuring a robust and comparative assessment of potential methodological approaches. **Table 1** provides details of the implemented architectures. The implemented lung cancer predictors can be further categorized into the ResNet family and efficient models.

**Table 1:** The summarization of the implemented deep learning-based lung cancer predictors in this research study.

Model types	Models	Params (M)
ResNet family	ResNet18	33.20
	ResNet50	46.20
	ResNet101	85.25
	ResNeXt101	47.53
	R2Plus1D	46.22
Efficient models	ShuffleNetv1	3.61
	ShuffleNetv2	3.41
	SqueezeNet	1.84
	MobileNetv1	12.88
	MobileNetv2	2.36
	R2Plus1D	46.22

Params refers to the number of parameters.

ResNet family: As a commonly used deep learning architecture for 3D vision applications, models from the ResNet family were originally designed with residual connections to overcome the gradient vanishing problem in deep learning models for image classification tasks. Later, the model structures were adapted to a wide range of 3D vision applications, such as video analysis, human action recognition, and medical image analysis. In this paper, we consider ResNet models with diverse depth configurations (ResNet18, ResNet50, ResNet101) (21–23), which impacts the trade-off between model performance and computational complexity. Additionally, an improved ResNet structure, ‘ResNeXt101’ (21–23), was considered as a predictor candidate due to its better capabilities introduced by a split-transform-merge strategy in the lower dimensional embeddings. Furthermore, R2Plus1D (22), a variant of ResNet with high performance in capturing spatial and temporal information, serves as a predictor candidate for verifying the transferability between video analysis and lung cancer prediction.

Efficient Architectures: Due to the high demand for computing resources, the application of deep learning-based diagnostic models has been limited. In this study, we included various efficient deep learning predictors (ShuffleNetV1 (23, 24), ShuffleNetV2 (23, 25), SqueezeNet (23, 26), MobileNetV1 (23, 27), and MobileNetV2 (23, 28)) to verify how data augmentation impacts the prediction performance of efficient models. ShuffleNetV1 is an efficient convolutional neural network (CNN) architecture that utilizes channel shuffle operations to exchange information among different group convolution branches. ShuffleNetV2 focused on more practical scenarios and introduced channel split operations for dense input/output while maintaining low computational complexity. SqueezeNet, one of the earliest compact CNN models, utilizes specially designed modules with squeeze and expand layers to achieve better efficiency trade-offs. MobileNetV1 is a widely used network structure in edge computing and embedded systems with depth wise separable convolutions, while MobileNetV2 is improved by introducing linear bottleneck layers and inverted residual blocks.

### Data augmentation methods for lung cancer predictors

In this subsection, we will categorize the data augmentation candidates into two categories: online and offline data augmentation. The key difference between them is the timing of performing the data augmentation. Online methods perform data augmentation while training the predictors; and the offline approaches perform data augmentation before the training phase.

Online data augmentation: As an essential tool for training deep learning models, online data augmentation has been widely applied in almost every computer vision task for decades due to its capability in augmenting data during the training phase. This allows effectively augmenting the existing dataset while suffering from relatively low quality in augmented data. Based on simple geometric and photometric transformations, researchers introduced automatic data augmentation strategy search methods, such as AutoAugment (29) and RandAugment (12). More recently, image mixing and random erasing-based approaches, such as Mixup (13), Cutout (15), and CutMix (14), have gained attention due to their high capabilities in computer vision tasks. Although these data augmentation approaches have proven their performance in various practical tasks, the transferability of their high capacity from traditional areas to medical domains remain ambiguous. In this work, we implement and adjust different online data augmentation methods from 2D image areas to 3D CT volume domains. Specifically, five methods were included: volume-based geometric data augmentation (V-GDA), volume-based RandAugment (V-Rand), volume-based Mixup (V-Mixup), volume-based Cutout (V-Cutout), and volume-based Cutmix (V-Cutmix).

- V-GDA: We included two common geometric transformations: RandFlip and RandRotate. The former randomly flips the CT volumes along a random axis with a probability of 50%. The latter randomly rotates the CT volume 90 degrees along a random axis with a probability of 50%.
- V-RA: We followed the algorithm and basic settings of RandAugment (12) while adapting the operations for 3D inputs and making them suitable for CT scans. The specific list of operations is shown in the **Supplementary Materials**.
- V-Mixup, V-Cutout and V-Cutmix: The original Mixup, Cutout, and Cutmix were designed for image classification tasks. To apply them to the lung cancer prediction task, we adapted the algorithms to enable them to accept 3D CT volumes.

The discovery cohort was rebalanced simply by oversampling the minority category then applied online data augmentation while the training stage.

Offline data augmentation: The rapid advancements in deep generative models have positioned offline data augmentation as a trending research topic across various fields. The core idea behind offline data augmentation is to augment an existing dataset prior to the training phase. These data augmentation algorithms are typically computationally intensive and thus impractical to use during the training stage. However, the offline augmentation approach allows for the generation of high-quality augmented data at the expense of computational efficiency. In this work, we utilize a diffusion model-based Med-DDPM (18) and a GAN-based HA-GAN (19) to evaluate the capability of SOTA deep generative models for data augmentation in lung cancer prediction. Specifically, these generative models are trained on the discovery cohort to generate synthetic lung nodule CT scans for data augmentation purposes. Unlike online data augmentation, which slightly modifies existing samples, deep generative model-based offline data augmentation synthesizes novel samples.

We assess these models in two tasks: rebalancing and incremental. In the rebalancing task, the generative models produce synthetic minority class samples, which are then mixed with real samples from the discovery cohort to rebalance the class distribution. For the incremental task, the generative models generate synthetic samples for both malignant and benign classes, which are then added to the rebalanced discovery cohort from the previous task. We generate additional samples amounting to 50%, 200%, and 500% of the original discovery cohort size, incorporating these as extra data. The case numbers for each task are summarized in **Table 2**. To demonstrate the performance improvement clearly, we also setup a baseline task that involved no data augmentation or oversampling cases.

**Table 2:** The summarization of the case numbers of the discovery cohort in different tasks.

Tasks	Malignant case number (Real/Synthetic or oversampled)	Benign case number (Real/Synthetic)	Total number
W/o DA + w/o oversampling (baseline)	49/0	101/0	150
Online DA + simple oversampling	49/52	101/0	202
Offline DA + Rebalance (RB)	49/52	101/0	202
Offline DA + 50% samples (Plus50)	49/102	101/50	302
Offline DA + 200% samples (Plus200)	49/252	101/100	502
Offline DA + 500% samples (Plus500)	49/552	101/500	1202

DA refers to data augmentation.

### Lung cancer prediction workflow

**Figure 2:**
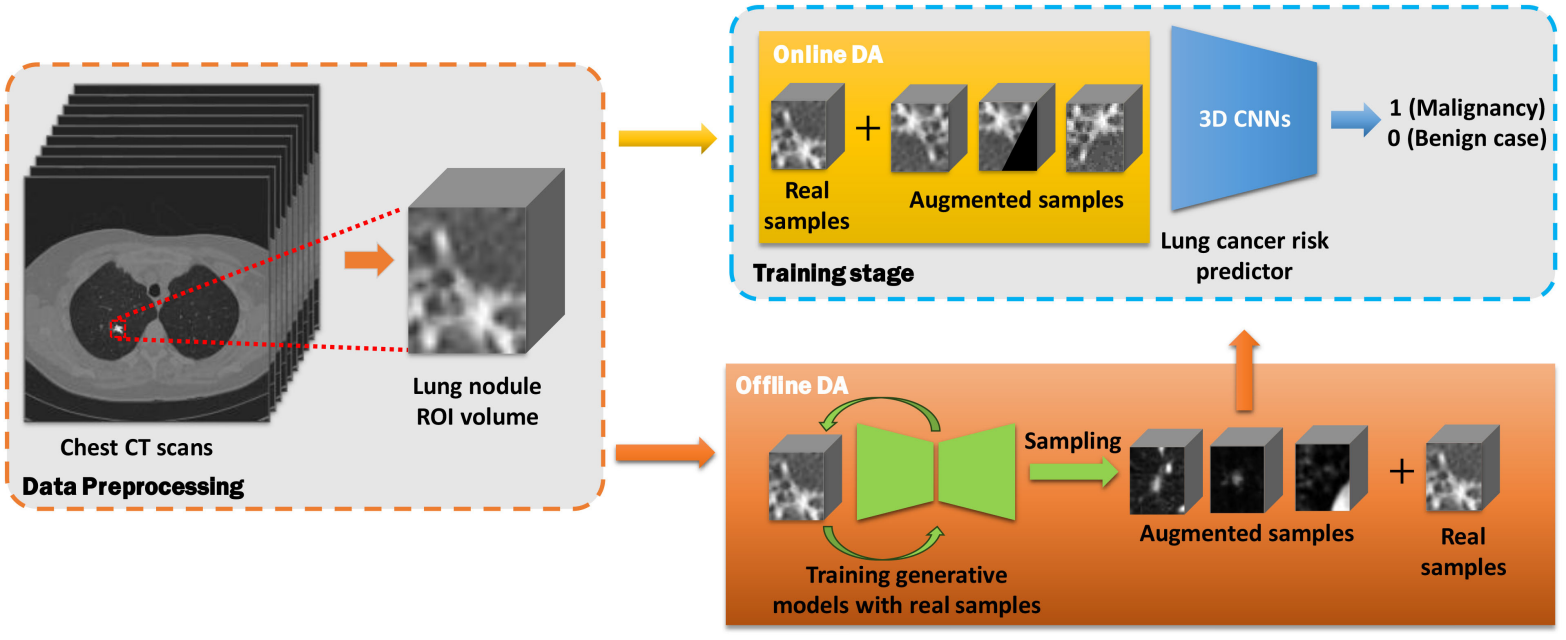
Workflow of online and offline data augmentation for lung cancer prediction using chest CT scans. The workflow is divided into three main parts: data preprocessing (orange dashed rectangle), data augmentation (yellow and orange blocks), and predictor training stage (blue dashed block).

The workflow for lung cancer prediction using online and offline data augmentation methods is illustrated in **Figure 2**. The entire pipeline can be divided into three main parts:

1. *Data Preprocessing:* Nodule region-of-interest (ROI) slices are extracted from chest CT scans in the discovery cohort based on segmentation annotations. These slices are then stacked into a three-dimensional CT volume.
2. *Data Augmentation:*
  - Online Data Augmentation: Augmented samples are generated from real samples during the training stage. Both real and augmented samples are used to train the prediction model.
  - Offline Data Augmentation: Real samples were first used to train generative models. The pre-trained generative models then synthesize augmented samples, which were then mixed with real samples for training the predictors.
3. *Prediction:* After the training stage, the lung cancer predictor classifies the lung nodule volumes into either malignant or benign cases using the validation cohort.

### Evaluation metrics

To comprehensively evaluate the candidate methods, we reported the performance of the developed models using three metrics: accuracy, F1 score, and area under the receiver operating characteristic (AUROC) score. Specifically, accuracy and F1 score were reported from the epoch with the highest AUROC score. Given that both online and offline data augmentation introduce randomness to the training stage, we reported prediction performance with a 95% confidence interval. For the online methods, each predictor was trained ten times using the same data augmentation settings and training set, with performance based on the results of these 10-fold repeated experiments. For the offline methods, synthetic samples were generated ten times to construct 10-fold training sets containing both synthetic and real samples. Performance was then reported from the predictors trained on these 10-fold training sets.

In order to provide a straightforward way for reviewing the quality of synthetic images, we applied the multidimension scaling (MDS) visualization to both real and synthetic samples. We firstly used a pretrained ResNet50 model to extract feature embeddings from 200 samples generated by each offline data augmentation method. For reference, we also extracted features from 200 real image samples. All feature embeddings were mapped into two-dimensional space using MDS.

### Implementation details

Lung cancer predictors were trained for 120 epochs with a learning rate of 1e-4. An Adam optimizer with parameters β_1_ = 0.9 and β_2_ = 0.99 is used for the training purpose. The batch size used for training the 3D models is 4. All the predictors are pre-trained by the Kinetics dataset. All experiments were run on the HPC of Université Laval using either an Nvidia Tesla V100 16GB or an Nvidia Tesla A100 40GB/80GB GPU.

## Results

### Clinical data description

The study population characteristics of discovery and validation cohorts were summarized in **Table 3**. There was no significant difference found between the discovery and validation cohorts regarding the study population characteristics. Participants from both discovery and validation cohorts have similar average ages, with p=0.195. As for sex distribution, the majority of participants were male, with 57.33% in the discovery cohort, compared to 54.37% in the validation cohort. Regarding race, the majority of participants are white in both cohorts (94.67% vs. 93.20%). The majority were current smokers in both cohorts, but the percentage was higher in the validation cohort than the training cohort (58.25% compared to 52.00%). In terms of the distribution of nodule location, the nodules in discovery cohort tend to locate at right lobe (58.00% vs. 53.40%) but tend to locate at left lobe (42.00% vs. 45.63%) in the validation cohort. The distribution of nodule sizes (<6 mm, 6-16 mm, ≥16 mm) shows no significant difference between the two cohorts, with similar percentages in each size range. The distribution of cancer type between the discovery and validation cohorts are close with a p=0.467. And the distribution of cancer stages was also similar between two cohorts, with small difference across the stages with a p=0.325.

**Table 3:** Study population characteristics of discovery and validation cohorts.

	Discovery cohort (N=150)	Validation cohort (N=103)	*P*
Age, mean (SD)	63.80 (5.05)	63.24 (5.03)	0.195
Sex, N (%)			0.321
Male	86 (57.33)	56 (54.37)	
Female	64 (42.67)	47 (45.63)	
Race, N (%)			0.900
White	142 (94.67)	96 (93.20)	
Non-white	6 (5.33)	96 (5.83)	
Smoking status, N (%)			0.164
Former	72 (48.00)	43 (41.75)	
Current	78 (52.00)	60 (58.25)	
Nodule location, N (%)			0.099
Right lobe	87 (58.00)	55 (53.40)	
Left lobe	63 (42.00)	47 (45.63)	
Nodule size			0.204
<6 mm	59 (39.33)	42 (40.78)	
6-16 mm	66 (44.00)	49 (47.57)	
≥16 mm	25 (16.67)	12 (11.65)	
Malignant cases	N=49	N=32	
Histology, N (%)			0.467
Small Cell Carcinoma, NOS	1 (2.04)	3 (9.38)	
Non-Small Cell Carcinoma, NOS	1 (2.04)	1 (3,13)	
Squamous Cell Carcinoma, NOS	6 (12.24)	4 (12.50)	
Adenocarcinoma, NOS	22 (44.90)	14 (43.75)	
Bronchiolo-Alveolar Adenoca	10 (20.41)	4 (12.50)	
Other or NOS	9 (18.37)	6 (18.75)	
Stage, N (%)			0.325
I	34 (69.39)	22 (68.75)	
II	5 (10.20)	3 (9.38)	
III	4 (8.16)	3 (9.38)	
IV	4 (8.16)	3 (9.38)	
NOS	2 (4.08)	1 (3.13)	

Continuous data were reported as mean ± standard deviation (SD), and categorical data as counts and percentages.

### Performance of baseline models

**Figure 3:**
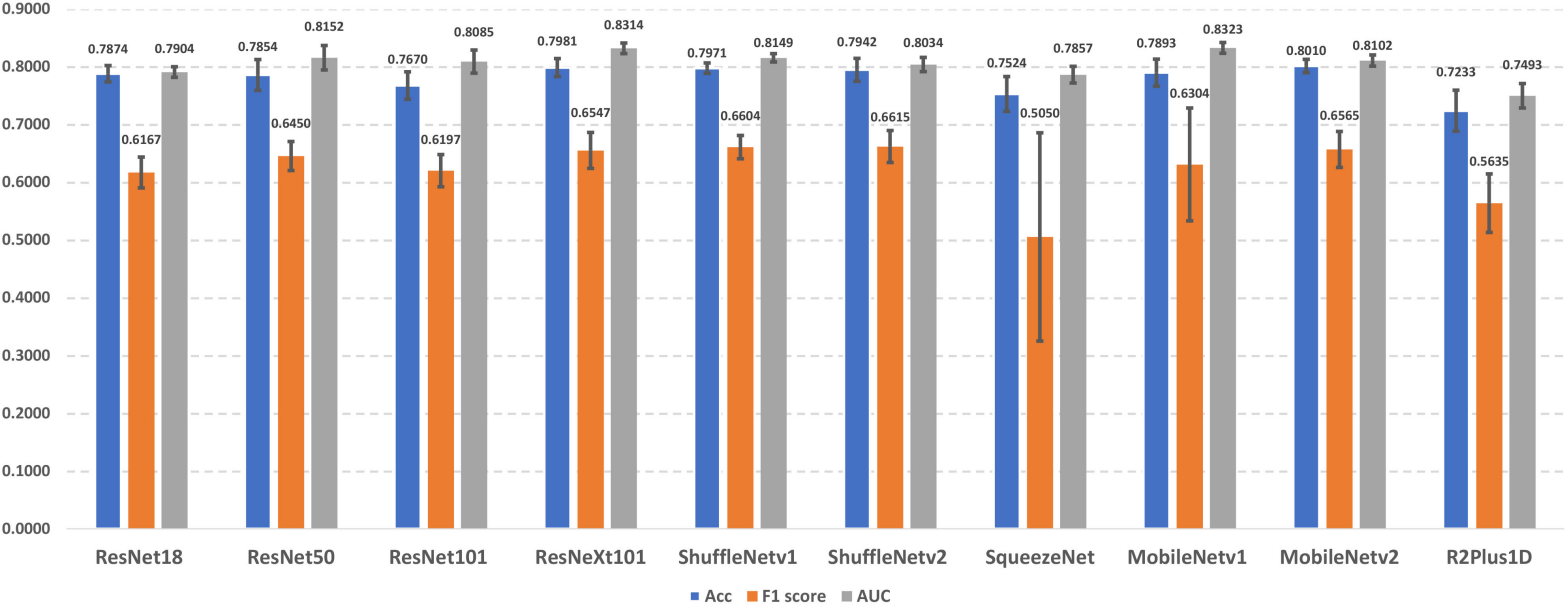
The demonstration of lung cancer prediction performance of baseline models.

In this section, we present the lung cancer prediction performance of candidate lung cancer predictors without using different data augmentation approaches (i.e., baseline models). The performance details were demonstrated in **Figure 3**. The experimental results indicate that MobileNetv2 achieves the best performance in accuracy (0.8010 ± 0.0115), and ShuffleNetv2 outperforms other predictors with 0.6615 ± 0.0276 F1 score. In addition, MobileNetv1 has the best AUROC score in 0.8323 ± 0.0093. On the other hand, R2Plus1D achieved the lowest accuracy (0.7233 ± 0.0355) and AUROC score (0.7493 ± 0.0210). The lowest F1 score (0.5050 ± 0.1802) is obtained by SqueezeNet. The performance among different predictors remains stable, which reflects their capability in lung cancer prediction. Notice that the F1 scores of SqueezeNet and MobileNetv1 are with large error bars, which indicates their sensitivity to certain data and potential overfitting problems.

### Performance of baseline models with online data augmentation methods

**Figure 4:**
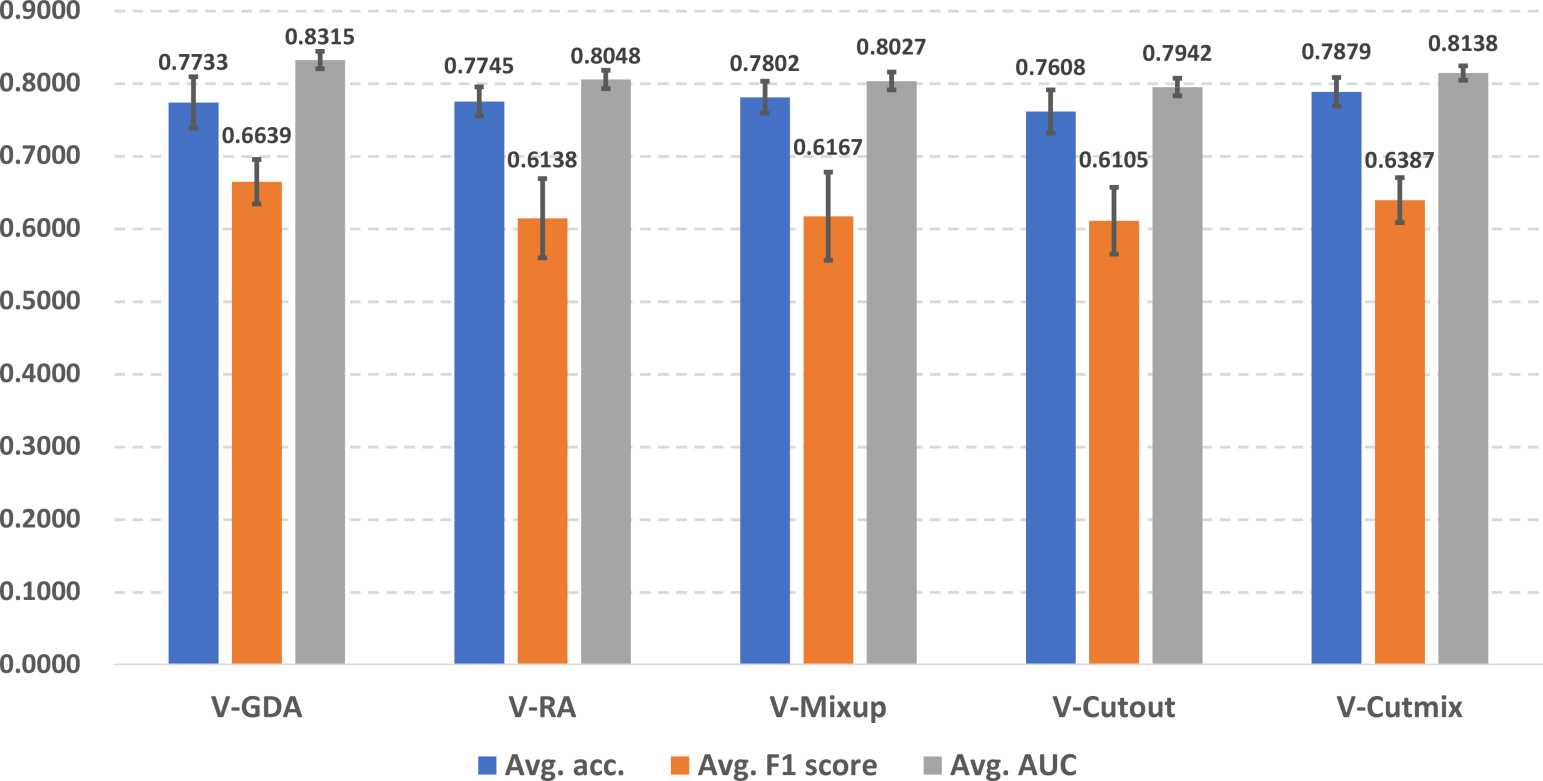
Illustration of online data augmentation performance in lung cancer prediction.

In **Figure 4**, we illustrate the online data augmentation performance in lung cancer prediction. The evaluation metrics were reported as average scores across ten predictors for each online data augmentation method. V-Cutmix achieved the best average accuracy (0.7879 ± 0.0196), while V-GDA showed the highest F1 score (0.6639 ± 0.0306) and the highest AUROC score (0.8315 ± 0.0119). On the other hand, V-Cutout has the lowest scores in all three metrics (accuracy: 0.7608 ± 0.0298, F1 score: 0.6105 ± 0.0460 and AUROC score: 0.7942 ± 0.0122).

The differences between the best and worst predictors are relatively small (about 2-5%), which suggests that all approaches have some merit, but some are slightly more suitable for the lung cancer prediction task than others. Different methods excel in different metrics, which indicates that the choice of online data augmentation technique might depend on which metric is most important for the specific scenario of lung cancer prediction in the clinical workflow.

### Performance of baseline models with offline data augmentation methods

**Figure 5:**
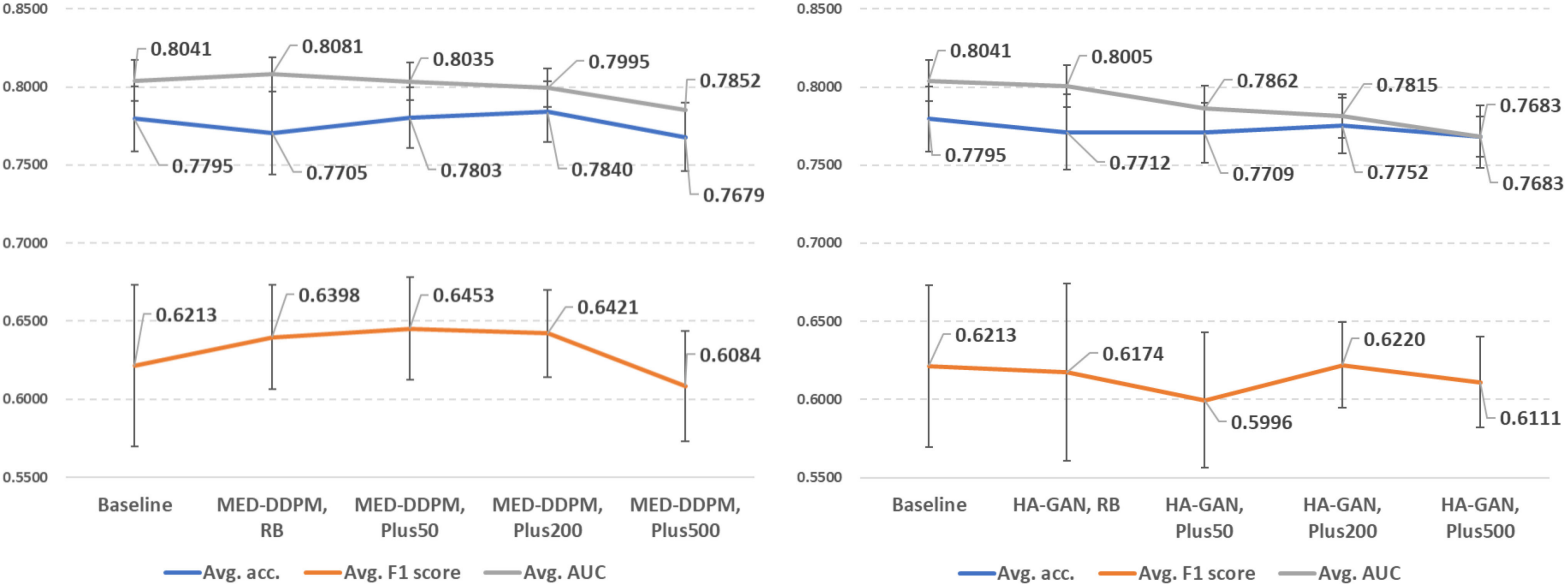
Demonstration of lung cancer prediction using offline data augmentation methods (left: MED-DDPM, right: HA-GAN). RB represents the rebalancing experimental setting, and PlusN indicates N% extra data is involved after the rebalancing.

In **Figure 5**, we demonstrate the offline data augmentation performance in lung cancer prediction. The 200% extra data from MED-DDPM after rebalancing have the best average accuracy (0.7840 ± 0.0195). The predictors trained with 200% extra data from MED-DDPM after rebalancing achieved the best average F1 score (0.6453 ± 0.0329). And the predictors trained with the training set rebalanced by MED-DDPM outperformed the other data offline augmentation settings in AUROC score (0.8081 ± 0.0109). Please note that that the predictors trained with synthetic data from HA-GAN have lower average performance in almost experimental settings compared to the baseline model.

The results suggest that the quality of synthetic data is more important than sheer quantity. MED-DDPM’s performance plateaus or even slightly decreases with 500% extra data, which indicates that there is an optimal point of data augmentation beyond which returns diminish. HA-GAN’s underperformance compared to MED-DDPM suggests that not all generative models are equally suited for medical data augmentation. This could be due to the potential overfitting problem that degrades the sample diversity from HA-GAN.

### Real vs. synthetic data comparison

**Figure 6:**
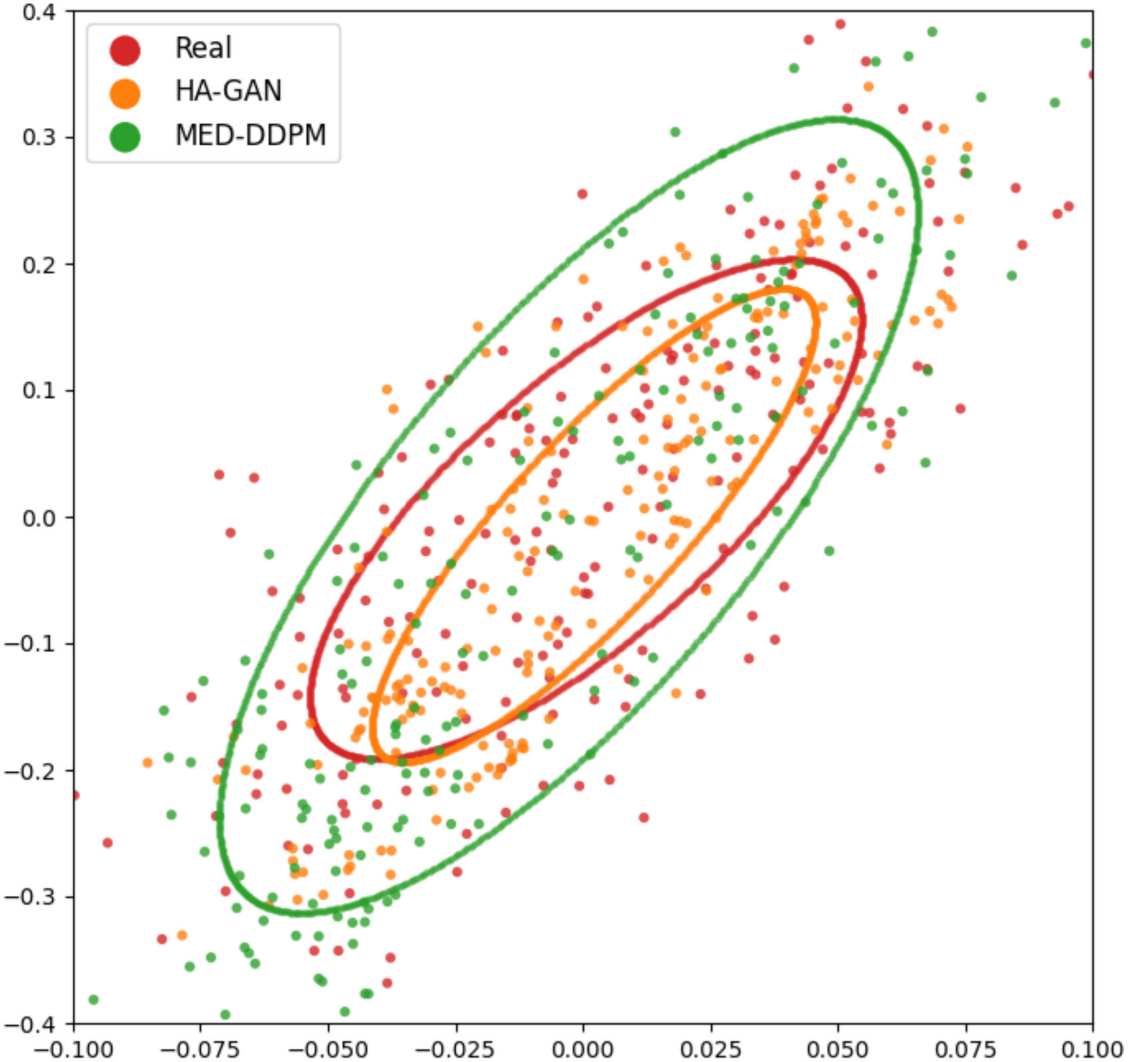
MSD visualization of offline data augmentation methods. Each point represents an image sample, with ellipses fitted to demonstrate the distribution of samples from different approaches. Red spots (enclosed in a red ellipse) represent real samples, while synthetic samples generated by MED-DDPM and HA-GAN are represented by orange and green spots within corresponding colored ellipses, respectively.

To evaluate the diversity and fidelity of synthetic data compared to real data, we followed HA-GAN (19) to use multidimensional scaling (MSD) to visualize the distance between real and synthetic samples. **Figure 6** illustrates the visualization results. MSD is a statistical technique that transforms high-dimensional data into a lower-dimensional space while preserving the relative distances between data points. In this study, MDS helps visualize the similarity and distribution of real and synthetic images that are embedded by a pretrained ResNet-50 model. The visualization suggests that MED-DDPM generates a more diverse range of augmented samples compared to HA-GAN, potentially capturing a broader spectrum of variations in the original data space. Conversely, HA-GAN synthesizes samples that more closely align with the distribution of real data. The experimental results highlight that two data augmentation methods were able to generate realistic samples, which are close to the real samples.

## Discussion

Data augmentation alongside deep learning approaches and has been developed and applied to various computer vision areas, such as image recognition (12–15), object detection (16), and medical imaging analysis (30). Although existing data augmentation approaches have shown promising results in the above applications, their performance in medical imaging analysis, especially lung cancer prediction, remains ambiguous and controversial. Moreover, previous studies (18, 19, 30) mainly focused on developing novel offline data augmentation methods but rarely compared the proposed methods to online data augmentation methods.

For online data augmentation, it is important to investigate if a more complex strategy approach actually benefits lung cancer prediction performance. According to **Figure 4**, compared to the baseline (no data augmentation applied), interestingly, V-GDA with the simplest strategy introduced the highest improvements in F1 and AUC scores, indicating that geometric transformation is an effective data augmentation approach. It excels by preserving the intricate anatomical structure and spatial relationships critical to medical imaging. Unlike aggressive augmentation strategies that can introduce artificial distortions or obscure diagnostic regions, RandFlip and RandRotate provide controlled variability that respects the fundamental integrity of lung tissue representations. Their effectiveness stems from simulating realistic imaging variations while maintaining the essential diagnostic features, making them particularly well-suited for nuanced medical image analysis where precision and anatomical fidelity are paramount. V-RA involves both geometric and photometric transformations, and randomly combines them and chooses magnitudes for them. The experimental results reveal that involving more transformations did not bring performance improvements and instead posed negative effects. Among the image mixing-based data augmentation methods, V-CutMix, as the technology combination of V-Mixup and V-Cutout, also took advantages from both and achieved improvements for all three-evaluation metrics. Our results highlight the significance of online data augmentation methods, which can achieve considerable performance improvements with relatively simple strategies. Furthermore, our findings indicate that involving more transformations or applying more complex strategies does not necessarily bring observable performance improvements.

We summarized the performance comparison of offline data augmentation methods in **Figure 5**. We observed that MED-DDPM offers improvements in F1 and AUC scores; however, it decreases accuracy in the rebalance subtask, achieving similar performance to V-GDA and V-CutMix. If we add an extra 50% and 200% training data from MED-DDPM, we observed improvements in accuracy and F1 score, allowing the performance to get closer to V-GDA and V-CutMix. However, neither MED-DDPM nor HA-GAN resulted in improvement across all three metrics. According to our results, the diffusion model-based MED-DDPM can achieve similar performance compared to first-class online methods with higher stability. However, the performance of HA-GAN highlights the importance of choosing a suitable offline data augmentation method, which can highly impact model performance of lung cancer prediction.

In addition, the number of synthetic samples generated by offline data augmentation methods impacts the model performance. Although synthetic data has been shown to improve diagnostic performance (30–32), the optimal number of synthetic samples appears to be context-dependent. To determine the appropriate proportion of synthetic data for lung cancer prediction, we illustrate the performance trends when introducing varying proportions of synthetic data into the original discovery cohort (**Figure 5**). We achieved the highest accuracy by adding 200% extra synthetic data from both MED-DDPM and HA-GAN, while performance fell below the baseline (0.7795) when adding 500% extra data samples. Regarding the F1 score, MED-DDPM reached its highest score when adding 50% synthetic data, and HA-GAN achieved its best F1 score with 200% synthetic data. However, when adding 500% extra synthetic data, the F1 scores of both methods dropped below the baseline (0.6213). The AUROC scores of both methods decreased as more synthetic data were introduced into the discovery cohort. The diminishing returns in synthetic data likely stem from two key factors: model saturation and data distribution limitations. As more synthetic data is added, the model’s learning gains plateau because the generated samples become increasingly redundant or less informative. In summary, 200% appears to be the optimal proportion of extra synthetic data to introduce into the discovery cohort for lung cancer prediction. The performance trends also highlight that too much synthetic data can negatively impact performance, with the optimal proportion varying depending on the generative models and specific tasks. Too much synthetic data can introduce noise or artificial variations that deviate from the underlying data distribution, potentially degrading model performance.

Furthermore, it is vital to understand that the online and offline data augmentation approaches address data shortage and imbalanced data problems in different ways. Online methods, due to their real-time nature, often use basic oversampling techniques and create slightly modified samples, tackling data shortage and imbalance simultaneously. Existing studies (33) suggest that online data augmentation may cause anatomical inconsistencies, as pixel-level transformations can produce irrelevant images that lack anatomical variation and disrupt the logic of medical image structure. However, our results provide a novel viewpoint, highlighting that some online data augmentation approaches can effectively mitigate these issues and yield significant performance improvements. Conversely, offline data augmentation typically employs generative models to model the data distribution of specific samples, then synthesize artificial data from these distributions. While some studies (30–32) have shown that online methods perform well in various medical tasks, our findings only partially support these conclusions. The performance of online data augmentation in lung cancer prediction is highly dependent on the proportion of synthetic data mixed into the discovery cohort. An improper proportion of synthetic data can lead to a noticeable reduction in performance, emphasizing the need for careful consideration of the synthetic data ratio.

There are two main factors that can be used to evaluate the synthetic samples’ quality: diversity and fidelity. In **Figure 6**, as for MED-DDPM, the synthetic samples appear to have a wider distribution than the real data. It captures the general shape and orientation of the real data distribution, at the same time, it overestimates the spread. In the case of HA-GAN, the samples have a distribution that more closely matches the real data. The HA-GAN samples’ distribution is slightly smaller and nestled within the real data distribution, which indicates that HA-GAN produces samples that are more realistic and closely aligned with the real data distribution. The apparent paradox in prediction performance can be explained by the trade-off between diversity and fidelity. MED-DDPM’s samples have greater diversity, despite lower average fidelity, provides more varied training examples. This helps the model learn more robust features and generalize better to unseen data. HA-GAN’s high fidelity indicates its capability in generating very realistic samples, but these may be too similar to the existing real data. While realistic, they don’t add much new information to improve the model’s predictive capabilities. The experimental results highlight the importance of choosing the offline data augmentation methods with a better trade-off between diversity and fidelity.

Our findings have significant clinical implications for lung cancer screening. By demonstrating improved model performance, the research addresses critical challenges in early detection, where traditional screening methods often struggle with limited data and imbalanced datasets.

The improvements in model performance directly translate to potentially clinical benefits:

- Enhanced Early Detection: The model performance improvement means more accurate identification of lung nodules at earlier stages, when treatment is most effective. Given that stage I lung cancer has an 81-85% survival rate compared to just 15-19% at stage IV, even marginal predictive improvements can significantly impact patient outcomes.
- Addressing Data Scarcity: The study provides a robust methodology for developing reliable predictive models with limited medical imaging datasets, a persistent challenge in healthcare AI. By demonstrating effective data augmentation techniques, the research offers a framework for developing more reliable diagnostic tools across resource-constrained settings.
- Generalizability: The comprehensive evaluation of multiple augmentation techniques provides clinicians and researchers a nuanced approach to developing machine learning models, emphasizing the importance of carefully selected data enhancement strategies.

Our research study has several strengths, including a comprehensive comparison and evaluation of modern data augmentation approaches for lung cancer prediction. Notably, our evaluation includes online data augmentation, which is often overlooked in recent studies. Our findings reveal that simple online data augmentation methods generally outperform offline methods, although the extent of performance improvement depends on the predictor types and the proportion of synthetic data used. These outcomes can guide more precise model and data augmentation selections in lung cancer prediction. However, our study has two significant limitations. First, the number of offline data augmentation candidates is limited due to the time-consuming training and sampling processes required for generative models. This limitation affects the reliability of performance evaluations for offline data augmentation. Second, the cohort scale is relatively limited. Due to the homogeneous backgrounds of participants, the sensitivity and generalization capability of data augmentation methods cannot be fully assessed. To address these issues, future research should include more generative model-based data augmentation approaches, conduct evaluations on larger, multi-institutional cohorts and consider diverse evaluation metrics for image quality, such as contrast-to-noise ratio (CNR) (34). In summary, data augmentation can be an effective tool for achieving better and more stable lung cancer prediction performance using CT scans. Considering the balance between performance and efficiency, online data augmentation is more suitable for this task due to its simpler strategy and efficient online training.

## Data Availability

NLST data presented in this study are publicly available through the TCIA archive. Related codes are available at https://github.com/Manem-Lab/DA-NLST.
