# Supplementary material for: Data augmented lung cancer prediction framework using the nested case control NLST cohort
Source: Front Oncol. 2025 Feb 25;15:1492758. doi: 10.3389/fonc.2025.1492758 (PMC11893409; doi:10.3389/fonc.2025.1492758)
Supplement: Supplementary file 1 [file SupplementaryFile1.docx]

**1. Data Augmentation Operations in the volume-based RandAugment (V-RA)**

To enhance the robustness of our model and mitigate overfitting, we employed a variety of 3D data augmentation techniques. These transformations were implemented using the MONAI library and were designed to introduce variability in the input data while preserving anatomical structures. Below, we provide details of the augmentations used in our study.

**1.1 Rotation Transformations**

Rotation transformations were applied along different axes to introduce variations in the orientation of the volumetric data:

- **Rotate X-axis**: Random rotation along the x-axis within a specified range.
- **Rotate Y-axis**: Random rotation along the y-axis.
- **Rotate Z-axis**: Random rotation along the z-axis.

**1.2 Translation Transformation**

- **Translation**: Random translation along all three spatial dimensions to simulate variations in positioning.

**1.3 Scaling Transformation**

- **Scaling**: Randomly scales the volume to introduce variability in size.

**1.4 Shearing Transformations**

Shearing distortions were applied to simulate anatomical deformations:

- **Shear X-axis**: Random shear along the x-axis.
- **Shear Y-axis**: Random shear along the y-axis.
- **Shear Z-axis**: Random shear along the z-axis.

**1.5 Intensity Transformations**

Adjustments to the intensity levels of the volumetric images were made to account for differences in imaging conditions:

- **Shift Intensity**: Randomly shifts the intensity values of the volume.
- **Adjust Contrast**: Adjusts the contrast by applying a random gamma correction.

**1.6 Histogram-Based Adjustments**

- **Equalization**: Applies histogram equalization to enhance contrast.
- **Solarization**: Inverts high-intensity values above a given threshold.
- **Histogram Shift**: Introduces controlled intensity shifts by modifying the histogram distribution.

**1.7 Sharpening Transformation**

- **Sharpening**: Enhances edge details in the volumetric images.
